# Characteristics and risk factors of rheumatoid arthritis in the United States: an NHANES analysis

**DOI:** 10.7717/peerj.4035

**Published:** 2017-11-24

**Authors:** Bei Xu, Jin Lin

**Affiliations:** Department of Rheumatology, The First Affiliated Hospital, College of Medicine, Zhejiang University, Hangzhou, Zhejiang, China

**Keywords:** Rheumatoid arthritis, National health and nutrition examination survey, NHANES, Risk factor

## Abstract

**Background:**

We examined the United States National Health and Nutrition Examination Survey (NHANES) database to determine factors associated with rheumatoid arthritis (RA) in adults 20 to 55 years of age.

**Methods:**

NHANES data collected between 2007 and 2014, excluding the 2011–2012 period, were used. Subjects were divided into those with and without RA. Demographic, clinical, and lifestyle factors were compared between the groups.

**Results:**

After applying inclusion/exclusion criteria, 8,789 persons were included in the study (8,483 without RA, 306 with RA). Multivariable analysis indicated that advanced age (odds ratio [OR] = 1.09, 95% CI [1.07–1.11], *P* < 0.001), regular smoking (OR = 2.19, 95% CI [1.49–3.21], *P* < 0.001), diabetes (OR = 2.00, 95% CI [1.35–2.95], *P* = 0.001), obesity (reference, normal or underweight; OR = 3.31, 95% CI [2.05–5.36], *P* < 0.001), and osteoporosis (OR = 3.68, 95% CI [1.64–8.22], *P* = 0.002) were positively associated with RA. Covered by health insurance (OR = 1.81, 95% CI [1.12–2.93], *P* = 0.016) and living in poverty (OR = 2.96, 95% CI [1.88–4.65], *P* < 0.001) were also associated with having RA. Mexican American, Hispanic white or other Hispanic ethnicity (reference, non-Hispanic white; OR = 0.54, 95% CI [0.31–0.96], *P* = 0.036), appropriate sleep duration (about 6–11 h, OR = 0.46, 95% CI [0.32–0.65], *P* < 0.001), and insufficient vitamin A intake (reference, recommended; OR = 0.70, 95% CI [0.50–0.98], *P* = 0.036) were negatively associated with RA.

**Discussion:**

Some factors associated with RA are potentially modifiable.

## Introduction

Rheumatoid arthritis (RA) is a chronic, autoimmune inflammatory disease with a female predominance, and is estimated to affect approximately 1% of the world’s population (Silman & Hochberg, 2001; Gibofsky, 2012). The etiology of RA is unknown, but genetic factors are associated with the condition and its severity (Silman & Hochberg, 2001; Begovich et al., 2004; Barton & Worthington, 2009; Gibofsky, 2012), and multiple environmental and lifestyle factors have been shown to be associated with its development (Silman & Hochberg, 2001; Begovich et al., 2004; Barton & Worthington, 2009; Gibofsky, 2012).

In RA, inflammation of the synovium leads to cartilage and bone destruction, with the joints of the hand and feet being the first affected (Lindhardsen et al., 2012; Ong et al., 2013). Other joints in the body are subsequently affected. Patients with RA are at increased risk for cardiovascular diseases, including atrial fibrillation and stroke, and mortality (Silman & Hochberg, 2001; Gibofsky, 2012), as well as other autoimmune diseases (Silman & Hochberg, 2001; Gibofsky, 2012). The association with cardiovascular diseases is of particular importance because the incidence of both conditions increase with age, and the world’s population is aging (Ong et al., 2013). The condition is extremely heterogeneous: it can wax and wane, be in remission for a long period of time and reoccur, or progress rapidly leading to debilitating joint destruction (Silman & Hochberg, 2001; Gibofsky, 2012).

There are a large number of disease-modifying antirheumatic drugs (DMARDs) and biological agents used to treat RA (Silman & Hochberg, 2001; Saag et al., 2008; Gibofsky, 2012). In some patients commonly used agents can result in rapid remission, while other patients will exhibit an inadequate response to multiple non-biological and biological agents (Silman & Hochberg, 2001; Gibofsky, 2012). Studying the effectiveness of different agents is somewhat hampered by the various classifications of disease severity and endpoints of treatment (Aletaha et al., 2010; Felson et al., 2011; Kelly, 2015).

Determination of modifiable risk factors, and treatment of comorbidities, may help prevent or delay the onset of RA, or improve treatment outcomes (Karlson, Van Schaardenburg & Van der Helm-van Mil, 2016; Turesson, 2016). While a large number of studies have examined risk factors for the development of RA, most are limited by patient number or geographic region. The National Health and Nutrition Examination Survey (NHANES) database of the Center for Disease Control and Prevention in the United States is an ongoing nationwide survey of the health of the United States population (Centers for Disease Control and Prevention, 2017a). As such, examination of its data offers a unique opportunity to determine disease prevalence and associations with other diseases and clinical and lifestyle factors.

Thus, the purpose of this study was to examine the NHANES database to determine factors associated with RA in adults 20 to 55 years of age.

## Method

### Data source and study population

Data from the National Health and Nutrition Examination Survey (NHANES) collected between 2007 and 2014, excluding the period from 2011 to 2012, were used for this analysis (Centers for Disease Control and Prevention, 2017a). Data from the period from 2011 to 2012 does not contain information regarding osteoporosis, one of the variables of interest in the current study; therefore, data from this period were not included in the analysis.

The NHANES program began in the early 1960s, and has been conducted as a series of surveys focusing on different population groups and health topics. The sample for the NHANES survey is selected to represent the United States population of all ages. Further information about background, design and operation are available on the NHANES website (http://wwwn.cdc.gov/nchs/nhanes). All of the NHANES data are de-identified, and analysis of the data does not require Institutional Review Board approval or informed consent by subjects.

Inclusion criteria for this analysis were participants between 20 and 55 years of age with complete outcome data of interest. Participants for whom no data on arthritis diagnosis, RA type, or weighting were available were excluded. For the analysis, participants were grouped into two groups: those with and without a diagnosis of RA. The outcome measure of the current analysis was the risk of developing RA.

### Study variables

#### Rheumatoid arthritis and other medical conditions

A diagnosis of RA was based on patient self-report as described in the NHANES Data Documentation, Codebook, and Frequencies (available at: http://wwwn.cdc.gov/Nchs/Nhanes/2001-2002/MCQ_B.htm#MCQ190). Briefly, in NHANES the diagnosis of RA was based on the following sequential questions: ‘Has a doctor or other health professional ever told you that you had arthritis?’, ‘How old were you when you were first told you had arthritis?’, and ‘Which type of arthritis was it?’.

A diagnosis of osteoporosis and diabetes were also self-reported based on interviewer-administered questionnaires. HIV status was based on laboratory data from the NHANES database.

Body mass index (BMI) was extracted from NHANES examination data. A BMI <18.5 kg/m^2^ was defined as underweight, between 18.5 and 24.9 kg/m^2^ as normal, between 25∼29.9 kg/m^2^ as overweight, and ≥30.0 kg/m^2^ as obese.

#### Demographic data

Age, sex, race/ethnicity, and marital status were recorded using interviewer-administered questionnaires from the NHANES database. Race/ethnicity was self-reported as Mexican American, Hispanic, Non-Hispanic White, Non-Hispanic Black, and Other Race (including multiracial). Marital status was self-reported as married, living with a partner, widowed, divorced, separated, and never married.

#### Behavioral factors

Smoking (cigarette/tobacco use), alcoholic drinking level, milk consumption, and sleep duration data were extracted from interviewer-administered questionnaires. As defined by the National Institute on Alcohol Abuse and Alcoholism (NIAAA), for women, heavy to high-risk drinking was defined as ≥4 or more drinks on any single day/occasion for ≥5 days in the past months. For men, it was defined as ≥5 drinks on any single day/occasion for ≥5 days in the past months. Moderate drinker was defined as up to one drink per day for women and up to two drinks per day for men. Occasional or non-drinker was defined as having less than 12 alcoholic beverages in any one year, OR, not meeting the definitions of heavy or moderate drinker.

Sleep duration was defined as follows. For young adults 18∼25 years old, 6∼11 h: recommended and may be appropriate; <6 and >11 h: not recommended (under or too much). For adults 26∼64 years old, 6–10 h: recommended and may be appropriate; <6 and >10 h: not recommended (under or too much).

Intake of caffeine was based on dietary interview data from the NHANES database, and categorized as none, recommended, and high. Milk consumption was categorized as never, regular, and irregular, and based on dietary interview data.

#### Dietary factors

Daily intake of total saturated fat (TSFA), vitamin A, B1, B2, B6, B12, C, D, E, K, niacin, folate, calcium, iron, magnesium, phosphorous, potassium, sodium, zinc, copper and selenium were extracted from dietary interview from NHANES database, and laboratory analysis. The recommended allowances as indicated by the 8th edition of the Dietary Guidelines for Americans are presented in Table 1*.*

**Table 1:** Recommended nutrient allowances as indicated by the 8th edition of the Dietary Guidelines for Americans.

Nutrients	Recommended allowances of nutrients
Total saturated fat intake	Female 20–30 years: ≤13.4 mg; >30 years: ≤12.3 mg
	Male 20–50 years: ≤16.7 mg; >50 years: ≤15.6 mg
Vitamin A	Female: 700∼3,000 mcg
	Male: 900∼3,000 mcg
Vitamin B1	Female: ≥1.1 mg
	Male: ≥1.2 mg
Vitamin B2	Female: ≥1.1 mg
	Male: ≥1.3 mg
Vitamin B6	Adults 20–50 years: 1.7∼100 mg
	Female > 50 years: 1.5∼100 mg
	Male > 50 years: 1.7∼100 mg
Vitamin B12	≥2.4 mcg
Vitamin C	Female: 75∼2,000 mg
	Male: 90∼2,000 mg
Vitamin D	15∼100 mcg
Vitamin E	15∼1,000 mg
Vitamin K	Female: ≥90 mcg
	Male: ≥120 mcg
Niacin	Female: 14∼35 mg
	Male: 16∼35 mg
Folate	400∼1,000 mcg
Calcium	20–50 years: ≥1,000 mg
	>50 years: ≥1,200 mg
Iron	Female 20–50 years: 18 mg
	Female > 50 years: 8 mg
	Male: 8 mg
Magnesium	Female 20–30 years: ≥310 mg
	Female > 30 years: ≥320 mg
	Male 20–30 years: ≥400 mg
	Male > 30 years: ≥420 mg
Phosphorous	≥700 mg
Potassium	≥4,700 mg
Sodium	≤2,300 mg
Zinc	Female: ≥8 mg
	Male: ≥11 mg
Copper	≥0.9 mg
Selenium	≥55 mcg
Caffeine	<300 mg

#### Socioeconomic status

Education level and family monthly poverty level index were extracted from the “Demographics variables and sample weights” data of the NHANES database. Demographic information was collected in the home prior to the health examination. A computer-assisted personal interviewing (CAPI) methodology was used. Insurance status was based on patient self-report.

### Statistical analysis

When examining data from the NHANES database, the analytical guidelines edited by the National Center for Health Statistics (NCHS) recommended using weighted analysis to assure national representation (Johnson et al., 2013). Weighting variables including pseudo-stratum (SDMVSTRA), pseudo-cluster (SDMVPSU), and dietary day 1 sample weight (WTDRD1) were used in all analyses. WTDRD1 was selected as the sample weight because dietary data were analyzed in this study. The weight used in the merged sample was revised to assure the national representation (Johnson et al., 2013). Age was summarized as mean ± standard deviation (SD). Categorical variables were summarized using a weighted proportion of people in the USA. The relations between parameters and RA were examined using survey-weighted logistic regression. Significant variables revealed by univariate analysis were subsequently analyzed by multivariate analysis. The significance level was set to 0.05. All analyses were performed with SAS statistical software (version 9.4, SAS Inc., Cary, NC, USA).

**Figure 1:**
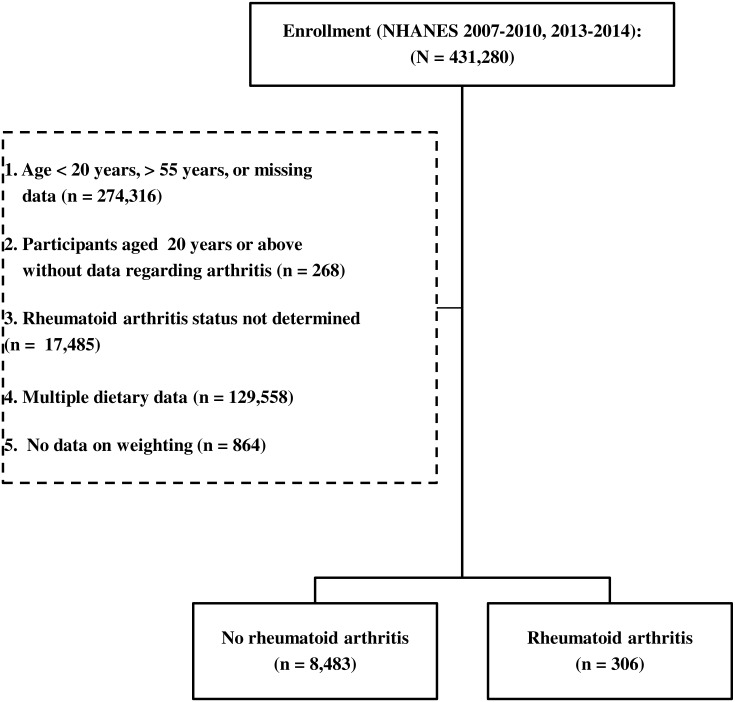
A flow diagram of participant inclusion.

## Results

### Study sample

The NHANES database collected between 2007 and 2014, excluding the 2011–2012 period, contained data of a total of 431,280 participants. Participants who were younger than 20 years or older than 55 years were excluded from the study. Participants for whom no data on arthritis diagnosis, RA type, or weighting were available were also excluded. Thus, the records of 8,789 persons met the inclusion criteria, and were separated into the without RA group (*n* = 8,483) and the RA group (*n* = 306). A flow diagram of participant inclusion is shown in Fig. 1.

The characteristics of the study sample using the recommended weighted analysis methods are shown in Table 2. The mean age of the participants without RA (36.4 ± 20.0 years) was significantly lower than the mean age of those with RA (44.6 ± 8.9 years). Relative to the without RA group, RA group had a higher proportion of females (61.0% vs. 49.5%), non-Hispanic white or black persons (84.3% vs. 74.3%), widow or separated/ divorced persons (25.3% vs. 11.9%), participants at or below the poverty level (69.1% vs. 51.1%), persons covered by health insurance (80.2% vs. 73.3%), regular smokers (57.6% vs. 38.3%), obese individuals (58.3% vs. 32.1%), persons having an inappropriate sleep duration (<6 h or >10 or 11 h, 28.7% vs. 12.8%), individuals with osteoporosis (5.7% vs. 0.8%) or diabetes (17.2% vs. 3.6%), and persons with an excess to toxic level of vitamin A intake (>3,000 mcg, 2.4% vs. 0.6%), a recommended sodium intake (≤2,300 mg, 30% vs. 22.1%), and an inadequate copper intake (<0.9 mg, 36.7% vs. 29.1%) (Table 2).

**Table 2:** Characteristic of study sample with and without rheumatoid arthritis.

	Without rheumatoid arthritis (*n* = 8,483)	Rheumatoid arthritis (*n* = 306)	*P*
***Demographic features***			
Age, years	36.4 ± 20.0	44.6 ± 8.9	**<0.001**
Sex			**0.003**
Male	4,214 (50.5)	110 (39.0)	
Female	4,269 (49.5)	196 (61.0)	
Education level			0.125
<9th grade	673 (4.5)	32 (7.0)	
9th–12th grade	3,290 (34.7)	134 (38.4)	
≥13th grade	4,510 (60.8)	140 (54.6)	
Race			**0.003**
Mexican American or other Hispanic	1,690 (11.5)	41 (7.6)	
Non-Hispanic white	3,420 (61.8)	157 (66.8)	
Non-Hispanic black	1,655 (12.5)	71 (17.5)	
Others	1,718 (14.2)	37 (8.1)	
Marital status			**<0.001**
Married/living with partner	5,092 (60.4)	165 (58.4)	
Widowed/divorced/separated	1,089 (11.9)	90 (25.3)	
Never married	2,297 (27.7)	51 (16.3)	
Family monthly poverty level index			**0.002**
≥3 (not poverty)	2,851 (48.9)	70 (30.9)	
<3 (poverty)	4,925 (51.1)	220 (69.1)	
Covered by health insurance	5,584 (73.3)	233 (80.2)	**0.044**
***Behavioral factors, lifestyle and health conditions***			
Regular smoking	3,223 (38.3)	171 (57.6)	**<0.001**
Level of drinking			0.067
Non-drinker	994 (11.4)	34 (10.1)	
Moderate	2,907 (39.1)	108 (49.5)	
Heavy	2,975 (44.8)	78 (34.9)	
Occasional	390 (4.7)	14 (5.6)	
Body mass index^a^, kg/m^2^			**<0.001**
<25	2,784 (35.1)	64 (19.5)	
25∼29.9	2,778 (32.9)	70 (22.2)	
≥30.0	2,880 (32.1)	169 (58.3)	
Appropriate sleep duration^b^	7,259 (87.2)	206 (71.3)	**<0.001**
HIV positive	41 (0.5)	2 (0.6)	0.718
Osteoporosis	55 (0.8)	15 (5.7)	**<0.001**
Diabetes	404 (3.6)	58 (17.2)	**<0.001**
Caffeine intake			0.474
None	1,367 (14.2)	40 (14.9)	
Recommended	5,958 (69)	203 (64.5)	
High	1,158 (16.7)	63 (20.6)	
Milk consumption			0.594
Never	2,043 (22.4)	68 (20.1)	
Regular	3,552 (43.3)	129 (47)	
Irregular	2,886 (34.3)	109 (32.8)	
***Dietary factors***^c^			
Higher total saturated fat intake	119 (1.4)	5 (1.3)	0.914
Vitamin A intake			**0.027**
Insufficient	6,524 (75.0)	231 (72.1)	
Recommended	1,907 (24.5)	72 (25.5)	
Excess (toxic)	52 (0.6)	3 (2.4)	
Inadequate vitamin B1 intake	2,698 (30.4)	108 (36)	0.112
Inadequate vitamin B2 intake	1,766 (18.3)	66 (19.8)	0.494
Inadequate vitamin B6 intake	2,503 (28.7)	115 (33.1)	0.130
Inadequate vitamin B12 intake	2,316 (26.3)	78 (22.4)	0.232
Inadequate vitamin C intake	3,186 (36.9)	103 (33.9)	0.454
Inadequate vitamin D intake	8,132 (95.6)	293 (95.7)	0.926
Inadequate vitamin E intake	8,407 (98.7)	304 (97.7)	0.448
Inadequate vitamin K intake	6,240 (72.1)	222 (70.7)	0.682
Niacin intake			0.676
Insufficient	1,626 (17.8)	58 (18.4)	
Recommended	4,953 (59.6)	188 (62)	
Excess	1,904 (22.5)	60 (19.6)	
Folate intake			0.065
Insufficient	3,442 (39.6)	135 (44.6)	
Recommended	4,239 (50.4)	150 (49)	
Excess	802 (10.0)	21 (6.4)	
Inadequate calcium intake	5,234 (59.3)	206 (61.8)	0.454
Inadequate iron intake	3,704 (42.1)	131 (40)	0.518
Inadequate magnesium intake	6,195 (72)	227 (73.2)	0.670
Inadequate phosphorous intake	941 (10.3)	44 (13.6)	0.103
Inadequate potassium intake	7,908 (93)	289 (92)	0.582
Excess sodium intake	6,486 (77.9)	213 (70.0)	**0.031**
Inadequate zinc intake	3,609 (41.2)	127 (42.6)	0.728
Inadequate copper intake	2,648 (29.1)	108 (36.7)	**0.018**
Inadequate selenium intake	1,045 (12)	49 (17.5)	0.059

**Notes.**

Age was presented as mean ± standard deviation, and other data were expressed as frequency (weighted %).

a Body mass index (BMI) <18.5 kg/m^2^ was defined as underweight, BMI between 18.5 and 24.9 kg/m^2^ as normal, BMI between 25 and 29.9 kg/m^2^ as overweight, and BMI ≥ 30.0 kg/m^2^ as obese.

b Appropriate sleep duration was defined as the time interval ranges from 6 to 11 h for people between 18 and 25 years of age, or from 6 to 10 h for people between 26 and 64 years of age.

c Recommended nutrient allowances are shown in Table 1.

### Factors associated with RA in persons 20 to 55 years of age

Univariate analysis indicated that advanced age, regular smoking, obesity, osteoporosis, diabetes, excess to toxic level of vitamin A intake, and inadequate copper intake were positively correlated with having RA. Living in poverty, being covered by health insurance, and being married were also positively associated with RA. On the other hand, male sex, Mexican American or other race, appropriate sleep duration, excess sodium intake, and inadequate copper intake were associated with a lower risk of RA.

Multivariable analysis indicated that advanced age (odds ratio [OR] = 1.09, 95% CI [1.07–1.11], *P* < 0.001), regular smoking (OR = 2.19, 95% CI [1.49–3.21], *P* < 0.001), diabetes (OR = 2.00, 95% CI [1.35–2.95], *P* = 0.001), and covered by health insurance (OR = 1.81, 95% CI [1.12–2.93], *P* = 0.016) were positively associated with having RA. Moreover, the odds of having RA was around 3 times higher for those living in poverty (OR = 2.96, 95% CI [1.88–4.65], *P* < 0.001) or who were obese (OR = 3.31, 95% CI [2.05–5.36], *P* < 0.001). Osteoporosis conferred an approximately 4-fold risk of having RA (OR = 3.68, 95% CI [1.64–8.22], *P* = 0.002). The increased risk of RA due to sex, marriage, excess sodium intake, and inadequate copper intake observed on univariable analysis was not present on multivariable analysis.

Factors associated with a lower risk of RA were Mexican American, Hispanic white, or other Hispanic ethnicity (OR = 0.54, 95% CI [0.31–0.96], *P* = 0.036), appropriate sleep duration (about 6–11 h, OR = 0.46, 95% CI [0.32–0.65], *P* < 0.001), and insufficient vitamin A intake (<900 mcg for males and <700 mcg for females; OR = 0.70, 95% CI [0.50–0.98], *P* = 0.036) (Table 3).

## Discussion

The purpose of this study was to examine a large population-based database to study factors associated with RA. Review of the NHANES database identified 8,789 persons eligible for inclusion in this analysis. The results indicated that advanced age, regular smoking, diabetes, and osteoporosis were associated with an increased risk of RA. On the other hand, Mexican American, Hispanic white or other Hispanic ethnicity, appropriate sleep duration, and insufficient vitamin A intake were associated with a lower risk of RA. Interestingly, the social factors of living in poverty and covered by health insurance were associated with increased risk of RA. While a population-based study such as this cannot examine cause and effect, many of the factors associated with the development of RA are potentially modifiable on an individual or societal scale.

**Table 3:** Univariate and multivariate analysis of factors associated with rheumatoid arthritis.

	Univariate		Multivariable	
	OR (95% CI)	*P*	OR (95% CI)	*P*
Age, years	1.09 (1.07, 1.1)	**<0.001**	1.09 (1.07, 1.11)	**<0.001**
Male	0.63 (0.46, 0.86)	**0.004**	0.71 (0.48, 1.04)	0.082
Education (Ref: <9th grade)		0.196		
9th–12th grade	0.71 (0.42, 1.19)			
≥13th grade	0.58 (0.32, 1.03)	0.0623		
Race (Ref: non-Hispanic white)				
Mexican American or other Hispanic	0.61 (0.39, 0.97)	**0.038**	0.54 (0.31, 0.96)	**0.036**
Others	0.53 (0.32, 0.88)	**0.014**	0.64 (0.37, 1.11)	0.113
Non-Hispanic black	1.3 (0.88, 1.92)	0.187	0.88 (0.56, 1.4)	0.595
Marital status (Ref: never married)				
Married/living with partner	1.64 (1.13, 2.39)	**0.010**	1.27 (0.77, 2.08)	0.344
Widowed/divorced/separated	3.62 (2.48, 5.28)	**<0.001**	1.41 (0.76, 2.61)	0.270
Poverty	2.14 (1.41, 3.25)	**<0.001**	2.96 (1.88, 4.65)	**<0.001**
Covered by health insurance	1.48 (1.01, 2.16)	**0.044**	1.81 (1.12, 2.93)	**0.016**
Regular smoking	2.18 (1.62, 2.95)	**<0.001**	2.19 (1.49, 3.21)	**<0.001**
Level of drinking (Ref: non-drinker)				
Moderate	1.44 (0.86, 2.41)	0.169		
Heavy	0.88 (0.48, 1.64)	0.698		
Occasional	1.34 (0.59, 3.07)	0.484		
Obesity (Ref: normal or underweight)				
Overweight	1.21 (0.85, 1.74)	0.293	1.29 (0.85, 1.94)	0.232
Obese	3.26 (2.21, 4.82)	**<0.001**	3.31 (2.05, 5.36)	**<0.001**
Appropriate sleep duration	0.37 (0.27, 0.49)	**<0.001**	0.46 (0.32, 0.65)	**<0.001**
HIV positive	1.34 (0.28, 6.42)	0.718		
Osteoporosis	7.13 (3.44, 14.77)	**<0.001**	3.68 (1.64, 8.22)	**0.002**
Diabetes	5.59 (4.09, 7.64)	**<0.001**	2.00 (1.35, 2.95)	**0.001**
Caffeine intake (Ref: none)				
Recommended	1.12 (0.72, 1.74)	0.616		
High	1.32 (0.82, 2.12)	0.250		
Milk consumption (Ref: regular consumption)				
Never	0.83 (0.57, 1.2)	0.322		
Irregular	0.88 (0.62, 1.25)	0.486		
Excess total saturated fatty acid intake	0.95 (0.34, 2.62)	0.916		
Vitamin A intake (Ref: recommended)				
Insufficient	0.92 (0.66, 1.28)	0.628	0.70 (0.50, 0.98)	**0.036**
Excess (toxic)	4.22 (1.01, 17.61)	**0.048**	1.74 (0.17, 17.37)	0.636
Inadequate vitamin B1 intake	1.28 (0.94, 1.75)	0.112		
Inadequate vitamin B2 intake	1.1 (0.84, 1.43)	0.494		
Inadequate vitamin B6 intake	1.23 (0.94, 1.6)	0.132		
Inadequate vitamin B12 intake	0.81 (0.57, 1.15)	0.234		
Inadequate vitamin C intake	0.87 (0.61, 1.24)	0.455		
Inadequate vitamin D intake	1.03 (0.51, 2.08)	0.927		
Inadequate vitamin E intake	0.55 (0.11, 2.66)	0.454		
Inadequate vitamin K intake	0.93 (0.66, 1.31)	0.682		
Niacin intake (Ref: recommended)				
Insufficient	0.99 (0.62, 1.6)	0.982		
Excess	0.84 (0.61, 1.15)	0.272		
Folate intake (Ref = recommended)				
Insufficient	1.16 (0.9, 1.48)	0.254		
Excess	0.65 (0.39, 1.1)	0.109		
Inadequate calcium intake	1.11 (0.84, 1.47)	0.455		
Inadequate iron intake	0.92 (0.7, 1.2)	0.518		
Inadequate magnesium intake	1.06 (0.81, 1.39)	0.671		
Inadequate phosphorous intake	1.37 (0.94, 2.01)	0.103		
Inadequate potassium intake	0.87 (0.53, 1.42)	0.582		
Excess sodium intake	0.66 (0.45, 0.96)	**0.031**	0.80 (0.47, 1.30)	0.348
Inadequate zinc intake	1.06 (0.76, 1.49)	0.728		
Inadequate copper intake	1.42 (1.06, 1.89)	**0.019**	1.31 (0.89, 1.95)	0.175
Inadequate selenium intake	1.55 (0.98, 2.45)	0.060		

**Notes.**

OR odds ratio CI confidence interval Ref reference group

The associations of RA with advancing age, smoking, and obesity are consistent with prior studies (Centers for Disease Control and Prevention, 2017b; Carlens et al., 2010; Turk, Van Beers-Tas & Van Schaardenburg, 2014; Versini et al., 2014; Jeong et al., 2017). The association of RA with age is well-known, with a peak onset among adults in their sixties (Centers for Disease Control and Prevention, 2017b). Why aging is associated with the development of RA is unclear, but current research suggests that immunosenescence that occurs with aging can lead to chronic inflammation and immune-mediated tissue damage (Weyand, Yang & Goronzy, 2014). The association of smoking and RA is also well-known. For example, a Swedish study consisting of a cohort of 277,777 male construction workers reported that ever-smoking was associated with increased risk of RA, with a relative risk (RR) = 2.1 (95% CI [1.7–2.5] (Carlens et al., 2010).

The exact pathophysiological mechanisms by which smoke results in RA are complex and have not been completely elucidated, but are known to involve increased oxidative stress, apoptosis (both increased and decreased depending on the cell type), development of a systemic proinflammatory state, development of autoimmune antibodies, and genetic factors (Chang et al., 2014).

Approximately 66% of persons with RA are obese, and apart from the destructive effect of excessive weight on already damage joints, fat affects the disease process (Versini et al., 2014). Excessive fat leads to greater production of inflammatory proteins that increase the joint inflammation due to the disease itself (Versini et al., 2014). Obesity and diabetes mellitus are related, and a population-based study in Korea also showed and association between diabetes and RA (Jeong et al., 2017). Our results indicated obesity conferred a 3-fold increased risk of RA. A recent meta-analysis that included 11 studies found that compared with individuals with a BMI under 30, obese individuals had a significantly increased risk of RA (RR = 1.25, 95% CI [1.07–1.45]) (Qin et al., 2015). Compared to normal weight subjects, the pooled RR for RA in obese individuals was 1.31, and in overweight individuals was 1.15.

The current analysis showed that osteoporosis was associated with increased risk of RA, which is consistent the data of earlier reports (Laan, Van Riel & Van de Putte, 1992; Haugeberg et al., 2000). We found that osteoporosis carried a 4-fold increased risk for RA. Avouac et al. (2012) studied 139 women with RA and 227 healthy women, and reported the prevalence of osteoporosis was 32% in patients with RA and 11% in the health controls and that age, osteoporosis, and low vitamin D level were independent risk factors of fractures in patients with RA. A study of 30,262 patients with RA and matched controls found that hip fracture risk was associated with >10 years’ duration of RA (RR = 3.4, 95% CI [3.0–3.9]), low BMI (RR = 3.9, 95% CI [3.1–4.9]), and use of oral glucocorticoids (RR = 3.4, 95% CI [3.0–4.0]) (Van Staa et al., 2006). Interestingly, an analysis of the NHANES database 1988–1994 cycle showed that femoral neck bone mineral density was similar between study subjects (aged 60 or above) with and without RA (Kinjo, Setoguchi & Solomon, 2007). It implied the association between osteoporosis and RA may be age dependent.

Hu et al. (2017) found no association of circulating carotenoid level and risk of RA in women using a nested case-control study design. It is of interest that insufficient vitamin A intake appeared to have a protective effect against RA, while on the other hand a toxic level of vitamin A was not associated with increased risk of RA on multivariate analysis. A similar study found that each increase in intake of 30 g fat fish (≥8 g fat/100 g fish) per day was associated with 49% reduction in the risk of RA (*P* = 0.06), whereas medium fat fish (3–7 g fat/100 g fish) was associated with significantly increased risk of RA; however, the author suggested that the result was likely due to chance because of a small sample size (Pedersen et al., 2005). The study found no associations between risk of RA and other dietary factors including intake of fruit and coffee, long chain fatty acids, olive oil, vitamins A, E, C, D, zinc, selenium, iron, and meat. A small cross-sectional case series of 53 women with RA indicated that the patients overall had a low dietary consumption of vitamins A and C, and zinc, and in the elderly in particular there was low consumption of vitamin E and selenium (Silva et al., 2014). In a survey study of a single-center RA registry, 24% of subjects reported that foods affect their RA, with 15% reporting improvement and 19% worsening (Tedeschi et al., 2017). Blueberries and spinach were the foods most often reported to improve RA symptoms, while soda with sugar and desserts were most often reported to worsen RA symptoms. A prior analysis of NHANES data indicated that bilirubin had a protective effect against RA (Fischman et al., 2010). The authors postulated the finding was because the anti-oxidant effects of bilirubin exert a physiological anti-inflammatory effect.

The association of appropriate sleep duration with lower risk of RA is an interesting finding, and is difficult to evaluate. It is possible the finding is a result of the fact that symptoms of the disease itself affect sleep, and that subjects without symptoms (without RA) naturally sleep for a more appropriate duration (Katz et al., 2016; Kim et al., 2016). Furthermore, the inter-relationships between sleep, depression, obesity, and physical inactivity in patients with RA are complex (Katz et al., 2016).

The associations between ethnicity and RA are similar to those previously reported, and most likely the result of genetic factors (Silman & Hochberg, 2001; Gibofsky, 2012). However, in the current study out of almost 9,000 participants, >300 had a diagnosis of RA. This is a prevalence of almost 3.5%, and is much higher than what is globally reported for RA. The reason for this result is likely because non-Hispanic white is associated with RA, and in our study population 40.7% were non-Hispanic white (19.7% for each: non-Hispanic black, Mexican American and other Hispanic, and other ethnicity).

Few studies have examined the influence of poverty and healthcare insurance on RA. Taiwan has universal health insurance that covers approximately 99% of the population. In a study of the Taiwanese health coverage database published in 2015, medical data of 23,900 RA patients from 2004 to 2008 were reviewed (Chen et al., 2015). Analysis of the data showed that the 5-year mortality rates were worse for patients with a low socioeconomic status than for those with a high socioeconomic status, even with a universal healthcare system in place.

There are a number of limitations to this study. Cause and effect cannot be determined in a population-based study such as this. While many of the factors associated with RA are potentially modifiable, identifying modifiable factors by comparing patients with prevalent RA to the general population by a cross-sectional study is not optimal. Many factors associated with RA may be likely consequences of disease rather than related to disease risk. For example, patients with RA are likely to have different diet and physical activity which might contribute to obesity. A prospective cohort study with incident RA as the outcome would be the optimal design for this type of analysis. Self-reported RA has relatively poor validity. In NHANES, however, the diagnosis of RA was based on the following sequential questions: ‘Has a doctor or other health professional ever told you that you had arthritis?’, ‘How old were you when you were first told you had arthritis?’, and ‘Which type of arthritis was it?’ Thus, we believe the validity of RA diagnosis is reasonable.

## Conclusion

The results of this NHANES database analysis indicated that advanced age, insurance, regular smoking, diabetes, obesity, and osteoporosis were positively associated with RA, while Hispanic white, Mexican American, or other Hispanic ethnicity, appropriate sleep duration, and insufficient vitamin A intake were negatively associated with RA. An interesting finding was that living in poverty and covered by health insurance were also positively associated with RA. Though cause and effect cannot be determined, modification of factors that are subject to change may help to reduce the risk of developing RA.

##  Supplemental Information

10.7717/peerj.4035/supp-1 Data S1 Raw data

## Additional Information and Declarations

### Data Availability

The following information was supplied regarding data availability: The raw data has been provided as Data S1.
